# Assessment of Serosal Invasion and Criteria for the Classification of Pathological (p) T4 Staging in Colorectal Carcinoma: Confusions, Controversies and Criticisms

**DOI:** 10.3390/cancers3010164

**Published:** 2011-01-04

**Authors:** Colin J. R. Stewart, Simon Hillery, Cameron Platell, Giacomo Puppa

**Affiliations:** 1 Department of Histopathology, SJOG Hospital, Perth, Western Australia; E-Mail: simon.hillery@sjog.org.au; 2 Colorectal Surgery Unit, SJOG Hospital, Perth, Western Australia and University of Western Australia; E-Mail: cplatell@cyllene.uwa.edu.au; 3 Division of Pathology, ‘G. Fracastoro’ City Hospital, Verona, Italy; E-Mail: gpuppa@yahoo.com

**Keywords:** colorectal, carcinoma, serosa, invasion, TNM, T4, stage, elastin, immunohistochemistry

## Abstract

Transmural spread by colorectal carcinoma can result in tumor invasion of the serosal surface and, hence, more likely dissemination within the peritoneal cavity and potentially to additional metastatic sites. The adverse prognostic significance of serosal invasion is widely accepted and its presence may be considered an indication for chemotherapy in patients with node negative disease. However, controversy persists regarding the most appropriate criteria for diagnosis and there are also practical difficulties associated with histological assessment in some cases. Therefore, serosal invasion may be under-diagnosed in a significant proportion of tumors, potentially leading to sub-optimal treatment of high-risk patients. The examination of multiple microscopic sections combined with ancillary studies such as cytology preparations, elastin stains, and immunohistochemistry may prove beneficial in selected problematic cases, but these are not used routinely. The relative prognostic significance of serosal invasion and of direct tumor spread to other organs, both of which are incorporated within the pT4 category of the AJCC/UICC TNM staging system, remains unclear. Further studies are required to demonstrate whether recent adjustments to the TNM staging of pT4 tumors are appropriate.

## 1. Introduction

Colorectal carcinoma (CRC) is one of the most common types of malignancy in most developed countries, accounting for approximately 10% of all new cancers and cancer-related deaths [1]. The introduction of screening for CRC has led to the detection of some tumors at an earlier stage and to the removal of potentially pre-malignant polyps [2]. However, screening programs are not uniformly available and most tumors still present symptomatically at which time many cases show invasion through the bowel wall with a significant proportion showing lymph node and/or distant metastases. Thus, the overall mortality rate remains 40–50%. Developments in molecular biology have led to a greater understanding of the pathogenesis of CRC and to some extent these advances have begun to have an impact at the clinical level, affecting surgical management and the choice of adjuvant therapies [3,4]. Nevertheless, the assessment of patient prognosis and the stratification of risk groups for post-operative therapy still rely primarily on traditional surgical-pathological staging.

**Figure 1.**
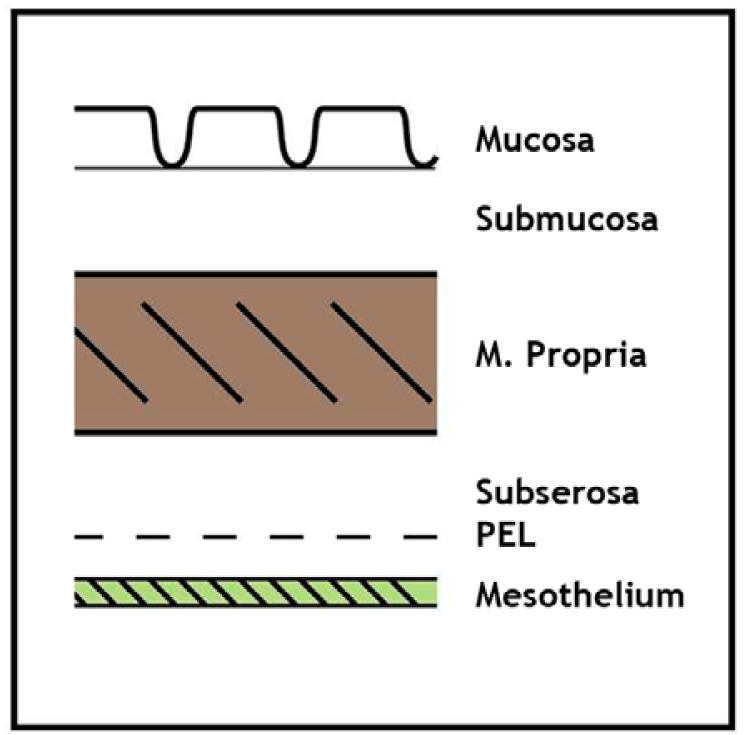
Schematic outline of the colorectal wall. M. propria: muscularis propria. PEL: peritoneal elastic lamina.

**Figure 2.**
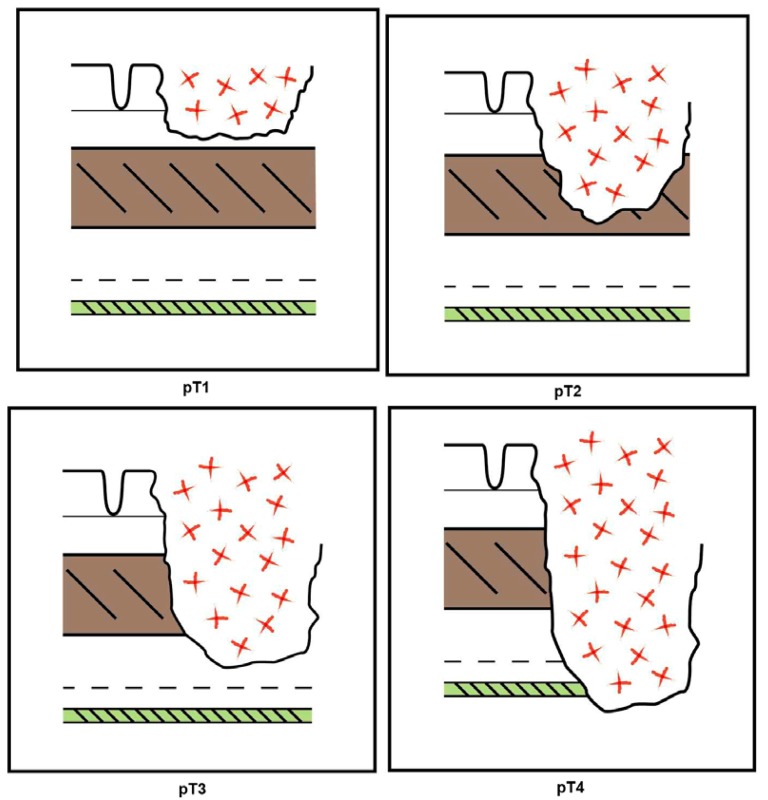
Schematic representation of the pathological substages (pT1 to pT4) of direct tumor spread.

**Figure 3.**
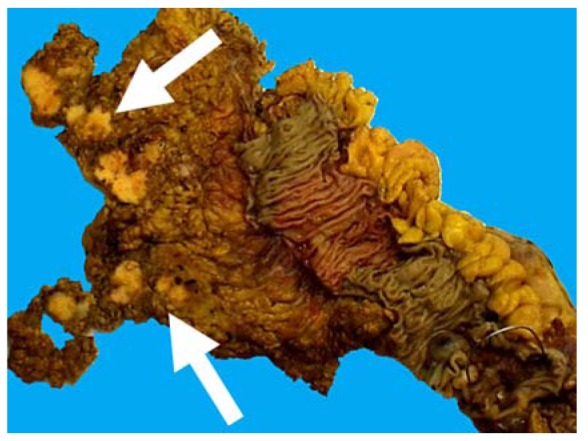
Macroscopic image of right hemicolectomy specimen showing multiple peritoneal tumor deposits (arrows).

Various staging systems for CRC have been developed and continue to be practiced [5] but probably the most widely used system is that based upon the local extent of direct tumor spread, the presence of regional lymph node metastasis, and the demonstration of distant metastasis, corresponding to the TNM categories, respectively, in the AJCC/ UICC classifications [6,7]. The pathological (p)T-category is further subdivided according to the extent of tumor spread within the bowel wall: pT1 corresponds to submucosal involvement, pT2 to invasion of the muscularis propria (muscle coat), pT3 to invasion of the subserosa or pericolic/ perirectal fat, and pT4, the most advanced local category, to tumor perforation, serosal invasion and/or direct extension into adjacent structures or organs. The anatomy of the bowel wall and schematic representations of these different tumor stages are depicted in Figures 1 and 2, respectively. It is now generally accepted that the pT4 category represents an adverse prognostic factor in patients with CRC independent of nodal status since transperitoneal invasion can lead to peritoneal carcinomatosis (Figure 3). Indeed, in one study, serosal invasion—one of the criteria for pT4—correlated more strongly with poor patient outcome than did the presence of lymph node metastasis [8]. The importance of serosal invasion and of direct invasion of adjacent structures has been recognized in the revised clinical stage groupings of patients with both node-negative and node positive tumors in the 7th edition of the TNM system [6,7]. Furthermore, although the role of adjuvant chemotherapy in patients with node negative (AJCC stage II, Dukes' stage B) CRC remains controversial, the presence of adverse risk factor such as pT4 status are used increasingly by oncologists as an indication for adjuvant chemotherapy [9-11]. Clearly, this makes the accurate histological assessment of serosal involvement and/or direct tumor invasion of other organs and structures critical. Nevertheless, difficulties and controversies persist regarding the diagnosis of serosal invasion and the categorization of the pT4 subgroup in colorectal neoplasia generally, and these issues are discussed in the current review.

## 2. The Normal Serosa

The serosa or peritoneum lines many structures within the abdominal cavity including the colon and rectum. The serosal investment is complete on those segments of the bowel that are suspended on a mesentery, that is the transverse and sigmoid colon, whereas the ascending and descending colon have a serosal surface antero-laterally but exhibit a non-peritonealized margin on their posterior aspect [7]. The peritoneum lines the anterior and lateral aspects of the proximal third of the rectum but only the anterior surface of the mid-rectum. The distal third of the rectum lies below the serosal reflection and therefore is extra-peritoneal. Thus, the potential of a colorectal neoplasm to involve a serosal surface depends not only upon the extent of local invasion, but also upon tumor location within the colon or rectum and its circumferential distribution within the bowel wall.

**Figure 4.**
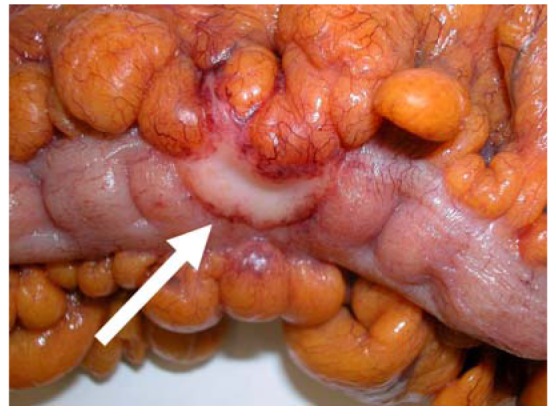
Macroscopic image of colon carcinoma with serosal involvement. Note the thickened central plaque of the tumor involving the serosa on the anti-mesenteric border (arrow) in comparison with the normal shiny serosal surface on either side.

Microscopically, the serosa is lined by mesothelial cells and their associated basement membrane, although the latter is not generally distinct on routine histological examination [12]. Underlying the mesothelium is a layer of fibrofatty stroma that constitutes the subserosa or the pericolic/perirectal tissue. The subserosa is of variable thickness but generally is most attenuated on the anti-mesenteric border of the bowel where tumors may rapidly involve the peritoneal surface after invading through the muscularis propria (Figure 4).

## 3. Problems in the Pathological Diagnosis of Serosal Invasion

**Figure 5.**
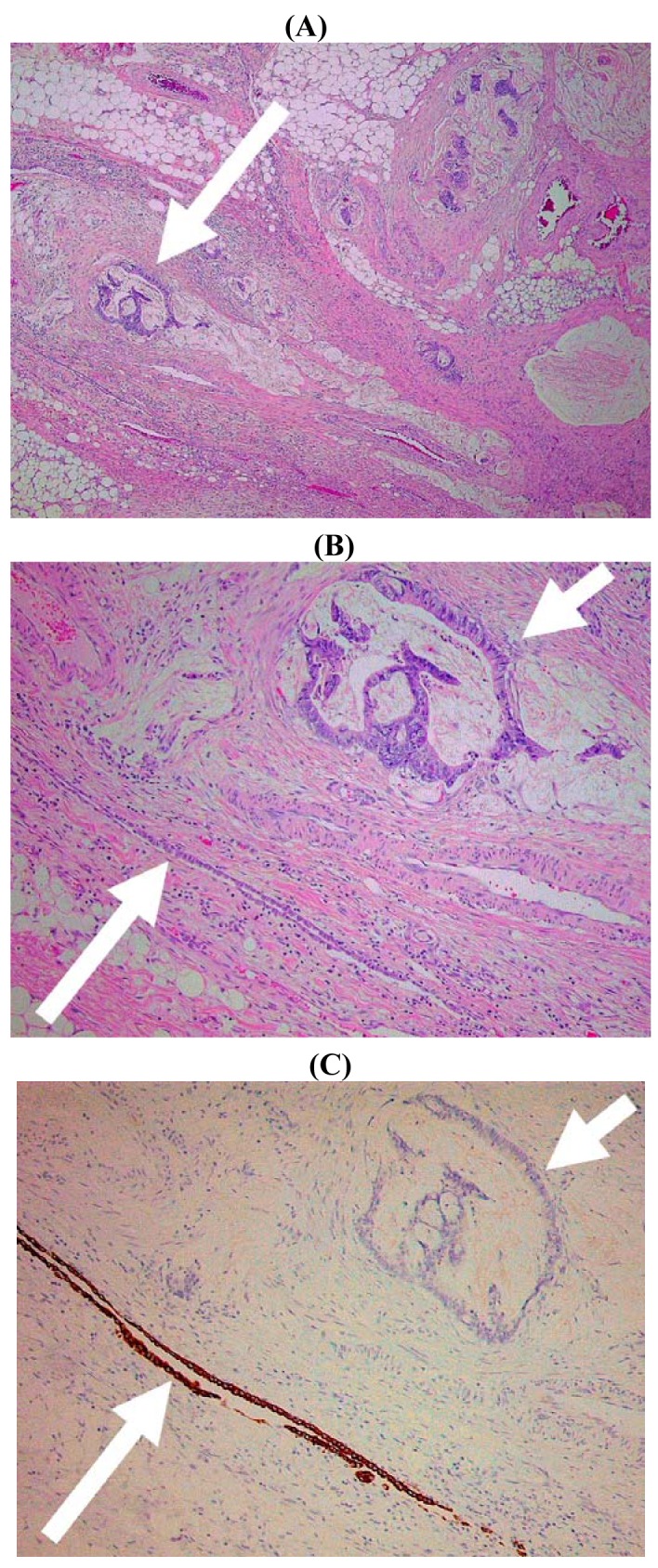
Histological image of colorectal carcinoma infiltrating the subserosal fat. (**A**) At low magnification there is no obvious relationship between the tumor and the serosal surface but note arrowed focus of malignant glands (×40). (**B**) At higher magnification the same tumor focus (short arrow) is adjacent to an inconspicuous serosal cleft lined by mesothelium (long arrow) (×100). (**C**) The mesothelium is highlighted by immunohistochemical staining for cytokeratin 7 (×100).

It might appear that the histological diagnosis of serosal invasion would be straightforward since this would be defined by transgression of the serosal surface (*i.e.*, the mesothelial layer) by malignant cells. Unfortunately several factors complicate such microscopic assessment. First, the mesothelial cells are relatively fragile and they may be stripped from the peritoneal surface during surgery, fixation, or post-operative handling. Therefore, in some instances it can be difficult to identify residual mesothelium overlying a tumor. Second, the normal peritoneal surface is not smooth and flat but follows the undulating contour of the pericolic/perirectal fat with clefts that invaginate from the external aspect of the bowel towards the muscle coat. Identification of these serosal clefts can be difficult, especially in the pathological setting, since they may be distorted by adjacent tumor infiltration or by inflammatory and fibrotic changes. Therefore, it is not always readily apparent that subserosal tumor cells are close to, or in continuity with, the peritoneal lining (Figure 5). This is an important issue since peritoneal clefts and areas of peritoneal reflection probably represent the most frequent site of serosal invasion by colorectal neoplasms [12-14]. Finally, and like many other types of carcinoma, CRC frequently elicit a prominent stromal reaction that includes fibroblasts/myofibroblasts, inflammatory and immune cells, proliferating vessels, and non-cellular matrix components. Indeed, these non-neoplastic elements are increasingly recognized to play an important role in cancer progression [15]. Even in the absence of direct tumor spread, these reactive stromal components can disrupt or efface normal anatomic structures including the peritoneum making it difficult to determine whether tumor cells have actually breached the serosal surface [16].

These diagnostic difficulties may partly explain the variation in the reported incidence of serosal invasion in CRC. For example, even in stage II (Duke's stage B) disease the rate has varied from 5–43% [17-22]. In one study, review of the original histology slides identified serosal invasion in 20/80 (22%) of stage II colonic cancers that initially had been reported as negative for this feature [23]. Conversely, a recent study that compared the reporting of histologic features in CRC found good agreement in the diagnosis of serosal invasion between four specialist gastrointestinal pathologists [24]. Nevertheless, studies from the Gloucester group, in which meticulous pathological examination of CRC specimens was performed, have shown consistently higher detection rates of serosal invasion than those reported in other centers and these have been shown to correlate with adverse patient outcomes [8,21,25]. These data suggest that careful macroscopic examination and histological sampling are critical to the accurate detection of serosal invasion.

## 4. Confusions in the Pathological Sub-Classification of Serosal Invasion

The different histological patterns of serosal invasion were classified in detailed studies by Shepherd and colleagues in which the authors described four patterns of local peritoneal involvement (LPI) [8,21,25]. Group 1 LPI was defined as tumor well clear of the closest peritoneal surface, group 2 LPI as mesothelial inflammatory/hyperplastic reaction with tumor close to but not actually present at the peritoneal surface, group 3 LPI as tumor present at the peritoneal surface with inflammatory reaction/mesothelial hyperplasia/‘ulceration’, and group 4 LPI as tumor cells demonstrated free in peritoneum and evidence of adjacent ‘ulceration’. These patterns have also been referred to as grades rather than groups [12]. The authors showed that LPI groups (or grades) 3 and 4 were associated with adverse patient outcomes whereas LPI groups 1 and 2 were not, and therefore only the former (LPI groups 3 and 4) were considered to represent ‘true’ serosal invasion.

These definitions have been very helpful in providing histopathologists with guidelines in assessing the presence or absence of serosal invasion in CRC (and other gastrointestinal malignancies), and they have been adopted as the basis for diagnosis in reporting protocols [26]. However, in our opinion the terminology has led to some confusion regarding what actually represents serosal invasion. The term ‘local peritoneal involvement’ appears to imply some degree of serosal invasion but, as noted above, LPI groups 1 and 2 were actually considered negative by Shepherd and colleagues [8,21,25]. However, this was not explicitly stated, possibly explaining the conflicting interpretation of peritoneal invasion by other authors. For example, earlier CRC reporting protocols and review articles that referenced the aforementioned studies stated that serosal involvement by CRC included three (rather than two) types of LPI [27-33]. These corresponded to the LPI groups 2-4 as originally defined by Shepherd and colleagues [8,21,25]; in other words, only tumor well clear of the serosal surface, or LPI group 1, was omitted. However, this was not clarified and, therefore, confusingly, peritoneal invasion types 1 to 3, as outlined in the protocols, equated to LPI groups 2 to 4. Furthermore, peritoneal invasion type 1 (tumor close to the serosal surface with inflammatory and/or mesothelial reaction, or LPI group 2), which was ‘negative’ according to the data provided by Shepherd *et al.* [8,21,25], was initially interpreted as positive for serosal invasion [27-29]. In subsequent protocols this issue become blurred since it was recommended that ‘the diagnosis of T4b (which defined serosal invasion at that time) encompass at least types 2 and 3 of serosal involvement’, in other words LPI groups 3 and 4 [30,31]. The most recent protocol from the College of American Pathologists is more definitive in that it recommends that ‘the T4a category (which now defines serosal invasion) ‘encompass types 2 and 3 of serosal invasion’ [26]. This approach seems more consistent with the original data of Shepherd *et al.* [8,21,25] but confusingly the text continues to state that ‘all the previously listed types of peritoneal involvement (*i.e.*, types 1-3 or LPI 2-4) were associated with decreased survival’ [26]. This comment is not supported by the references provided in the protocol and, to our knowledge, has not been substantiated in subsequently published work. Indeed, a study of stage II colonic carcinomas by Lennon and colleagues showed that only the presence of free lying tumor cells overlying the mesothelium (LPI group 4) was significantly associated with adverse prognosis [18]. These authors suggested that the diagnosis of serosal invasion should be limited to such cases, a view shared by Douard *et al.* [34]. Therefore, pathologists have been faced with a dilemma. Does serosal invasion include LPI groups 2 to 4, LPI groups 3 and 4, or only LPI group 4? It seems likely that the application of different criteria has contributed towards the variations in the reported incidence of serosal invasion in the literature noted earlier.

Leaving aside the confusion surrounding the varied interpretation of the LPI criteria, in our experience the most common practical dilemma when applying the LPI system is in separating tumors that are ‘close to’ but do not involve the serosa (LPI 2) from those that invade the peritoneum (LPI 3). This is not an academic issue since this distinction separates negative from positive cases, at least according to the original data of Shepherd and colleagues [8,21,25]. One problem is that the cut-off value for ‘close to’ has never been defined [35], although it has been proposed that invasion within 1 mm of the free peritoneal surface should be recorded as an ‘indication of possible transperitoneal spread’ [36]. However, as far as we are aware no data are available to determine whether this finding is associated with an increased risk of recurrence, and therefore, it is unclear at present how useful this information would be in determining patient prognosis and optimal management.

## 5. Value of Ancillary Studies

The assessment of serosal invasion in CRC depends upon careful macroscopic examination and appropriate sampling followed by thorough histological examination including the examination of multiple sections if necessary [14,32]. Perhaps surprisingly, ancillary studies have made relatively little contribution towards accurate diagnosis in this problematic area. However, three techniques have been explored that might provide useful additional information. The first method relies upon the examination of cytologic material rather than conventional histological tissue sections. Cellular material from suspicious areas of the bowel surface can be obtained using scrape or imprint techniques and these have demonstrated tumor cells in 9–26% of CRC [37-39]. The detection of malignant cells using these cytology preparations often correlates with positive histological assessment of serosal invasion, but not all cases demonstrate both findings suggesting that these methods may have a complementary role in diagnosis. A related cytological approach utilizes peritoneal washings to detect ‘free’ intra-peritoneal tumor cells, similar to the more widespread use of this technique in the staging of gynecological malignancies. It should be noted that the presence of malignant cells in peritoneal fluid could be a consequence of tumor metastases to lymph nodes or other sites and therefore would not necessarily be the result of direct trans-serosal spread by the primary tumor. The clinical significance of positive peritoneal cytology in patients with CRC has been debated since the results of studies are partly conflicting, but one meta-analysis concluded that positive cytology was an adverse prognostic factor [40].

**Figure 6.**
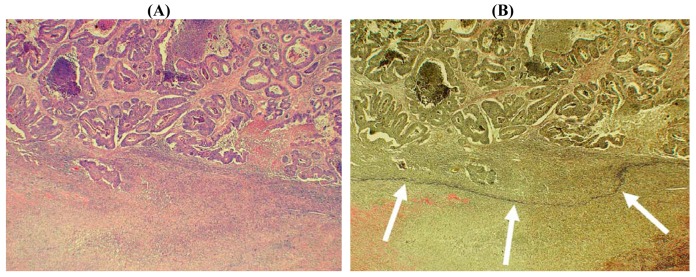
Histological image of colorectal carcinoma invading the subserosal fat. (**A**) The conventional hematoxylin and eosin stain shows tumor glands (upper field) with a marked reactive stroma towards the serosal surface (lower field). The relationship of the tumor to the native serosa is not clear. (×40) (**B**) Elastin stain shows an intact peritoneal elastic lamina (arrows) deep to the malignant glands suggesting that the tumor has not penetrated the serosal surface (×40).

A second approach to the diagnosis of serosal invasion utilizes elastin stains to highlight the peritoneal elastic lamina (PEL). Normally, the PEL comprises a relatively delicate layer of elastic fibers that lie just deep to the mesothelium [41], shown schematically in Figures 1 and 2. The importance of the PEL in pathological situations such as neoplasia is that it could provide a surrogate anatomical marker in those cases where tumor destruction or prominent fibro-inflammatory changes have distorted and effaced the native serosa (Figure 6) [16]. It should be noted that since the PEL is not considered to represent part of the normal serosa, the presence of tumor cells close to (or even penetrating) this structure does not necessarily equate with serosal invasion. Nevertheless, tumor extension beyond the PEL does provide indirect evidence of possible serosal invasion. This interpretation is supported by two studies in which CRC exhibiting extra-mural invasion were examined using elastin stains. Shinto *et al.* subdivided a series of pT3 CRC (that is cases considered negative for serosal invasion on routine histologic examination) into those with ‘shallow’ invasion and those with ‘deep’ invasion corresponding to malignant infiltration superficial to, or beyond, the PEL, respectively [42]. Deep invasion was an independent adverse prognostic factor. More recently, Kojima and colleagues studied peritoneal elastic lamina invasion in 564 pT3 and pT4 cancers [43]. Elastic lamina invasion correlated with other adverse histologic parameters and was an independent risk factor for recurrence in patients with stage II colonic malignancies.

In our experience, one limitation of elastin staining is that it is not always possible to clearly demonstrate the PEL in all cases of CRC. Although this could be a result of extensive tumor infiltration, it is also possible that inflammatory changes might lead to destruction of the PEL. There also appear to be areas in which the PEL is less distinct, as noted by Knudson [41]. Previously, we found that immunohistochemistry was no more sensitive than a conventional elastin stain (Miller's method) in demonstrating the PEL [44], but it is possible that different histochemical stains may demonstrate increased sensitivity in this area. For example, Kojima and colleagues were unable to demonstrate the elastic lamina in only 10 of 564 cases in their series [43]. It is also noteworthy that the application of elastin staining to appropriate tumor samples could be used to demonstrate both extra-mural vascular (venous) invasion and PEL invasion, thereby highlighting two independent and important adverse prognostic factors in patients with CRC [45-48].

**Figure 7.**
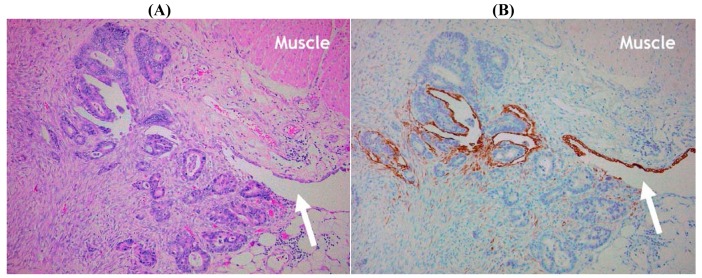
Histological image of colorectal carcinoma with serosal invasion on the anti-mesenteric border. (**A**) Note proximity of the muscle (muscularis propria, upper right field) to the serosal surface (arrow) (×100). (**B**) Immunohistochemistry for cytokeratin 7 highlights the mesothelial cells and confirms tumor invasion of the serosal surface (×100).

The final technique that might prove helpful in the evaluation of potential serosal invasion is based upon the immunohistochemical demonstration of mesothelial cells. Immunohistochemistry has the advantage of offering relatively cell-type specific labeling and, therefore, in theory could highlight residual mesothelium (and hence the serosal surface) when this is inconspicuous using routine hematoxylin and eosin (H&E) staining. Several ‘mesothelial markers’ including calretinin, D2-40/podoplanin, WT1 and cytokeratin (CK) 5/6 are used routinely in diagnostic histopathology practice, but in our experience CK7 staining is often most useful in this context since it labels mesothelial cells strongly whereas reactive stromal elements and tumor cells in the majority of CRC are negative. We have found that CK7 staining is particularly useful in demonstrating the deep serosal clefts that are easily overlooked on routine staining, and the presence of tumor cells invading and disrupting the mesothelium confirms the presence of serosal invasion (Figures 5 and 7). However, to our knowledge no studies to date have formally assessed the value of mesothelial cell immunostaining as an adjunct marker in the assessment of CRC.

One general problem with ‘enhanced pathologic analysis’ in CRC (encompassing, for example, the examination of multiple microscopic sections or use of special staining methods) is that the additional studies are labor-intensive and time consuming. Accordingly, these techniques are not universally recommended or applied in routine histopathological analysis.

## 6. Controversies in the Substaging of pT4 Tumors

As previously noted, the histopathologic criteria for assigning CRC to the category of most advanced local spread (pT4) includes direct invasion of adjacent structures or organs as well as serosal invasion. In earlier reporting protocols and guidelines [27-33,49], and in a related TNM commentary [50], these have been substaged pT4a and pT4b, respectively. One potentially confusing aspect of this classification has been the appropriate assignment of tumors that have directly invaded other structures via a serosal surface (for example, a cecal carcinoma adherent to and infiltrating the wall of the sigmoid colon). It is possible that some such cases may have developed initially on the basis of benign adhesions with subsequent tumor penetration across a central inflammatory interface [13,51]. Thus, since there would be no direct involvement of a free serosal surface and minimal risk of peritoneal dissemination, such tumors could be classified as pT4a. Conversely, tumors may initially involve the serosal surface with later contiguous invasion into another structure as a result of malignant adhesions. These cases would be at risk of peritoneal spread even if there was no apparent free serosal surface involvement at the time of surgery. As noted by in the guidelines issued by the U.K. Royal College of Pathologists, such tumors would fulfill criteria for both pT4a and pT4b substages [49], but presumably, by convention, would be assigned to the higher of the stage groupings (*i.e.*, pT4b). We believe that it is impossible to distinguish which of these potential mechanisms of invasion has occurred by pathologic examination of an en bloc tumor resection but it has been our policy to classify such cases as pT4b.

**Figure 8.**
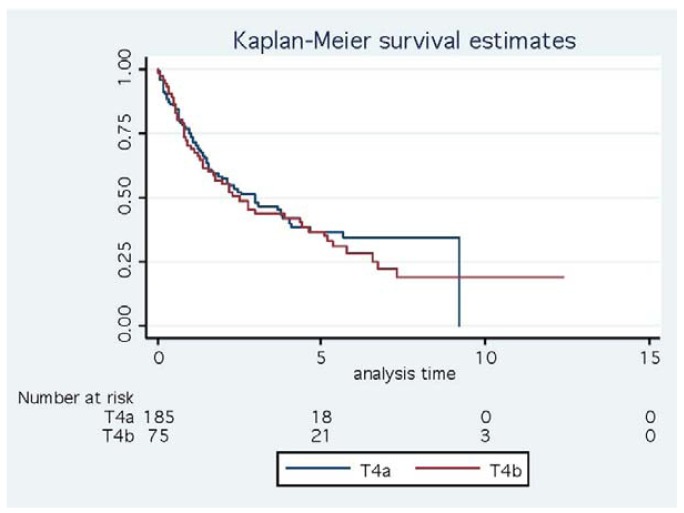
Survival analysis of 260 patients with pT4 colorectal carcinoma. Note that the pT4a and pT4b cases have similar outcomes (pT4a - direct invasion of other structures, pT4b - serosal invasion).

The basis for the pT4 subdivision has also been called into question. We have argued previously that the evidence supporting the initial pT4a/4b split was limited in that it appeared to be based upon unpublished data and the purported differences in patient outcomes were stated to be statistically not significant [52]. Furthermore, analysis of pT4 CRC cases in our own department has shown very similar survival curves for these subgroups (Figure 8). Nevertheless, the earlier staging classification was at least consistent with multiple studies identifying serosal invasion as an adverse prognostic factor in CRC, provisionally meriting its designation as the more advanced tumor stage (T4b). In the 7th edition of the AJCC/TNM classification there has been an alteration to the substaging of pT4 tumors with reversal of the previously recommended T4a and T4b categories [6,7]. In other words, pT4a now defines tumors that involve or penetrate the visceral peritoneum whereas direct invasion of other organs or structures is classified as pT4b. Surprisingly, this staging alteration was made without any initial comment or explanation [52,53]. Subsequently, the basis for these changes was stated to be a study (at that time available only in Abstract form) that demonstrated a 10-20% worse outcome for tumors showing direct invasion of other organs compared to serosal invasion in both lymph node negative and positive CRC categories [54].

In our opinion, several important issues are raised by this modification of the pT4 subsets. These partly relate to the validity of the new pT4 stages and partly to the way that changes to the TNM staging of CRC staging have been made. On the first issue, the rationale for inverting the order of the pT4a/pT4b subsets was based upon two recently published complementary studies that used Surveillance, Epidemiology, and End Results (SEER) population-based data on more than >130,000 patients with colonic cancer and >35,000 patients with rectal cancer [55,56]. The advantage of such studies is self-evident in that they draw upon data obtained from multiple centers over extended time periods (1992–2004) and thus comprise very large patient populations for statistical analysis. However, some limitations of these data must be considered, at least as far as the accuracy of staging information is concerned, since it is unlikely that the standard of pathological assessment was uniform either between different centers or over the recruitment time interval of these series. Potential problems related to the changing interpretation of tumor deposits/lymph node metastases during the study period have also been highlighted recently [57]. As discussed previously, the histological assessment of serosal invasion (new pT4a) has received appropriate emphasis in the pathology literature only recently, and studies from the U.K. and North America in the past 10 years have demonstrated sub-optimal reporting of this finding [58,59]. Although the use of reporting protocols and proformas has improved the proportion of reports that include key adverse prognostic data (such as serosal invasion), these by themselves do not necessarily indicate optimal tumor sampling and microscopic examination. In the SEER studies, only 13.7% of colonic carcinomas and 8.5% of rectal tumors were classified as pT4, and the corresponding figures for serosal invasion (new pT4a) were only 7.8% and 3.5%, respectively (data derived from Table 1 [55] and Table 2 [56], respectively). Unfortunately in the new staging system it is not possible to determine what proportion of pT4b tumors (showing direct invasion of other organs) were also positive for serosal invasion, but even if the majority of cases were so-classified the reported incidence in the SEER studies is still low. In comparison, it has been proposed that serosal invasion should be identified in 30% of colon cancers and 10% of rectal cancers [60], while the U.K. Royal College of Pathologists suggest as a quality measure that *at least* 20% of unselected colonic tumors should be positive for serosal invasion [49]. Meticulous pathological examination in one center recorded serosal invasion in 59% of colonic cancers and 26% of rectal cancers [8,25]. Thus, it is likely that the figures for pT4, particularly pT4a, reported in the SEER studies represent a significant under-diagnosis of serosal invasion and this could influence prognostic associations in the same way that inadequate lymph node sampling may lead to under-staging. This would also explain the apparently high proportion of pT4 tumors showing direct invasion of other organs (T4b/T4a ratio). While many studies do not provide separate data for the subsets of pT4 tumors, serosal invasion usually is reported to be at least 2-4 times more common than direct invasion of other organs [17,21,22,51,52,61]. However, according to the SEER data tumors demonstrating direct invasion of other organs or structures (new pT4b) account for 44% of all pT4 colon cancers and 59% of all pT4 rectal cancers (data derived from Tables 1 [55] and 2 [56], respectively), and this represents a surprisingly high proportion of such cases.

These issues illustrate a further limitation of both the previous and the revised versions of the TNM system since the pT4 category of CRC has included varied patterns of invasion that potentially have different clinical significance [36]. In the earlier TNM classifications, because stage pT4b included tumors showing involvement of the free serosal surface and also tumors showing direct invasion of other organs and structures across a serosal surface, it was not possible, based upon the stage category alone, to separate the clinical outcomes associated with these two processes. Using the new staging categories, pT4b includes all CRC that directly infiltrate adjacent organs regardless of whether this occurs via the serosa or through a non-peritonealized margin (the latter may be exemplified by a carcinoma of the ascending colon infiltrating the posterior abdominal wall, or a low rectal tumor infiltrating the prostate). Since such cases are now ‘lumped together’ with those tumors invading other organs across a serosal surface, it will not be clear from the staging data which of these processes is more important in terms of prognosis. As outlined in Table 1, it is also important to note that direct comparisons between the earlier and the revised pT4 substages are not possible by simply ‘inverting’ the groupings, since the representative constituents of these groupings are not consistent. Thus, it is critical that future studies carefully specify which staging systems are applied so that inappropriate comparisons of incompatible data are not made. We believe that it would have been better if the criteria for pT4 designation were documented and substaged separately since this would permit comparison with historic studies and eventually provide the prospective data from which the relative prognostic significance of the different invasive patterns could be determined.

Leaving aside the potential limitations of the pathologic data in the SEER studies, and the suboptimal information provided by the pT4 substages, in our opinion the process of revision of TNM staging in CRC has also been questionable. The changes to pT4 classifications were premature since they did not follow best practice in evidence-based medicine upon which changes to worldwide tumor staging should be based. First, alterations to staging were made initially on the basis of unpublished data which were not subject to peer review. Second, although the data have since been published, they are yet to be supported by an independent data set, at least in regard to the subdivision of pT4 stages (the data from the National Cancer Database and the Rectal Pooled Analyses presented in the SEER studies [55,56] do not appear to include information on T4 subsets). Indeed, in a recent Japanese study, TNM7 did not provide optimal stratification of patient survival [62] Third, the data appeared inconsistent with the earlier studies suggesting that serosal invasion was the more adverse prognostic factor in pT4 tumors, as repeatedly stated in earlier reporting protocols and TNM commentaries. While the latter assumption was based upon limited data, as noted above, one would expect that apparently conflicting findings would be subject to even greater scrutiny and independent confirmation prior to any stage alteration. In this regard, it is noteworthy that TNM 6 and TNM 7 have been subject to considerable criticism on the way that they have altered the pathological assessment of pericolic tumors deposits and lymph node metastases [63,64]. For these reasons, TNM 6 and 7 have not been adopted within the U.K. and other European countries.

Cancer staging systems are not static and indeed they must continue to evolve to the benefit of all patients with neoplastic conditions including CRC. However, changes to staging have major implications for patient management and they may make comparisons of data problematic or impossible. We agree with the sentiments of Quirke and colleagues that alterations to TNM staging must be evidence-based and therefore reflect high-quality and independently verified data [63,64].

## 7. Conclusions

Accurate macroscopic and microscopic examination of surgically resected CRC specimens is essential to reliably determine tumor staging and patient prognosis. Increasingly, the presence of adverse prognostic factors such as pathologic substage pT4 disease is considered an indication for adjuvant chemotherapy in lymph node-negative cases. Serosal invasion by tumor cells represents one of the criteria for pathological stage (p)T4 disease but the histological identification of this feature is not always straightforward since normal anatomic landmarks including the serosal surface may be obscured during tumor invasion or as a result of tumor-associated fibro-inflammatory changes. These practical difficulties in diagnosis have been aggravated by inconsistency in the interpretation of criteria for serosal invasion. Ancillary techniques may prove helpful in problematic cases but these have not been studied in detail and at present they are not used routinely. The recently revised TNM system for CRC (TNM 7) has adjusted previous guidelines for the substaging of pT4 tumors such that serosal invasion is classified as pT4a whereas direct invasion of other organs or structures is classified as pT4b. However, the basis for this alteration is questionable and further studies using high-quality and independently verified data are required to determine the optimal substaging of CRC.

## Figures and Tables

**Table 1.** Comparison of pT4 substage distribution of earlier and revised TNM classifications for 10 hypothetical colorectal carcinomas showing invasion of the free serosal surface (n = 6), trans-serosal invasion of other organs or structures (n = 3), and non-serosal direct invasion of other organs or structures (n = 1).

**Type of invasion**		**Earlier guidelines**		**TNM 7**	
		**4a**	**4b**	**4a**	**4b**
Free serosal surface	n = 6	0	6	6	0
Trans-serosal invasion of other organs	n = 3	0	3	0	3
Non-serosal invasion of other organs	n = 1	1	0	0	1
Total	n = 10	1	9	6	4
